# Comorbidities and clinical outcomes in adult- and juvenile-onset Huntington’s disease: a study of linked Swedish National Registries (2002–2019)

**DOI:** 10.1007/s00415-022-11418-y

**Published:** 2022-10-18

**Authors:** Hannah Furby, Suzanne Moore, Anna-Lena Nordstroem, Richard Houghton, Dimitra Lambrelli, Sophie Graham, Per Svenningsson, Åsa Petersén

**Affiliations:** 1 Roche Products Ltd, Welwyn Garden City, UK; 2 F. Hoffmann-La Roche Ltd, Basel, Switzerland; 3 Evidera, London, UK; 4 Section of Neurology, Karolinska University Hospital, Stockholm, Sweden; 5 Translational Neuroendocrine Research Unit, Department of Experimental Medical Science, Medical Faculty, Lund University, Lund, Sweden

**Keywords:** Huntington's disease, Movement disorders, Comorbidities, Disease burden, Mortality, Epidemiology, Real-world evidence

## Abstract

**Background:**

Huntington’s disease (HD) is a rare, neurodegenerative disease and its complex motor, cognitive and psychiatric symptoms exert a lifelong clinical burden on both patients and their families.

**Objective:**

To describe the clinical burden and natural history of HD.

**Methods:**

This longitudinal cohort study used data from the linked Swedish national registries to describe the occurrence of comorbidities (acute and chronic), symptomatic treatments and mortality in an incident cohort of individuals who either received the first diagnosis of HD above (adult onset HD; AoHD) or below (juvenile-onset HD; JoHD) 20 years of age, compared with a matched cohort without HD from the general population. Disease burden of all individuals alive in Sweden was described during a single calendar year (2018), including the occurrence of key symptoms, treatments and hospitalizations.

**Results:**

The prevalence of HD in 2018 was approximately 10.2 per 100,000. Of 1492 individuals with a diagnosis of HD during 2002 and 2018, 1447 had AoHD and 45 had JoHD. Individuals with AoHD suffered a higher incidence of obsessive–compulsive disorder, acute psychotic episodes, pneumonia, constipation and fractures compared with matched controls. Individuals with JoHD had higher incidence rates of epilepsy, constipation and acute respiratory symptoms. Median time to all-cause mortality in AoHD was 12.1 years from diagnosis. Patients alive with HD in Sweden in 2018 displayed a pattern of increased clinical burden for a number of years since diagnosis.

**Conclusions:**

This study demonstrates the significant and progressive clinical burden in individuals with HD and presents novel insights into the natural history of JoHD.

**Supplementary Information:**

The online version contains supplementary material available at 10.1007/s00415-022-11418-y.

## Introduction

Huntington’s disease (HD) is a rare, genetic, neurodegenerative and ultimately fatal disease [1, 2] caused by a cytosine adenine guanine (CAG) trinucleotide repeat expansion in the huntingtin gene, resulting in the production of toxic mutant huntingtin protein [1–3]. The disease is characterized by cognitive, behavioral and motor symptoms and signs, leading to progressive functional decline and loss of independence [2, 4]. Clinical diagnosis of HD is defined by the onset of unmistakable motor signs, such as chorea, that typically occur between the ages of 30 and 50 years, although the onset of motor symptoms can also occur earlier (juvenile-onset HD [JoHD]; ≤ 20 years) or later (late-onset HD; > 60 years) [2]. HD has an average prevalence of approximately 8–10 per 100,000 [5–8], of which between only 1 and 9.6% of all individuals will have JoHD [9]. Many HD mutation carriers already have cognitive changes and psychiatric symptoms, such as depression, apathy and irritability, in the premotor (premanifest) stage of the disease which precedes a clinical diagnosis.

Compared with individuals with adult-onset HD (AoHD), those with JoHD typically have a longer CAG repeat expansion and a distinctly different clinical profile. Non-motor signs including cognitive and behavioral problems typically manifest first, but unlike the characteristic Huntington’s chorea symptom that is typical of individuals with AoHD, rigidity, bradykinesia and dystonia are more severe in people with JoHD [10]. Furthermore, epileptic seizures also tend to be much more frequent [11]. The rate of clinical decline is often faster in people with JoHD, attributable to the larger CAG repeat expansion in these individuals [12]; however, the clinical profile is still not well characterized due to small sample sizes and short observation times. Disentangling pathophysiological and epidemiological differences between JoHD and AoHD is important for the successful treatment of people with HD [13].

Treatment options for HD remain limited and, with no disease-modifying therapies currently available, clinical care is focused on disease management by alleviating symptoms and maintaining the quality of life [14] through a combination of approaches, including pharmacotherapies, physiotherapy, occupational health, psychotherapy and dentistry among others [14]. Comorbidities are known to complicate HD management, though comparatively little research has been undertaken to compare the burden of comorbidities in individuals with HD with the general population, or to describe the rate of comorbidities following diagnosis. It is not easy to tease apart comorbidities and clinical symptoms that occur during the course of HD. For example, individuals with HD also experience a high psychiatric burden, with a reported lifetime prevalence of 33–76% [15]. The occurrence of depression is a core behavioral sign of HD. In addition to being part of the behavioral neuropathology of HD, the disease also has its own specific life stressors due to individuals struggling with relatives affected by the illness and the issues related to presymptomatic genetic testing [16]. Several studies have described the burden of HD in a routine care setting; however, these studies have typically placed an emphasis on healthcare resource use and associated economic burden, often over shorter time periods, with less emphasis on the elevated incidence of other comorbidities and symptoms associated with HD [17–22].

This study aims to describe the burden and natural history of HD, by assessing the incidence of comorbidities (including acute and chronic conditions), symptomatic treatments and mortality in individuals with HD between 2002 and 2019 using Swedish national registries. Specifically, this analysis seeks to: (1) compare incidence rates (IRs) of comorbidities (i.e. additional conditions co-occurring with HD), clinical events (i.e. outcomes directly caused by HD) and symptomatic therapies in incident patients (i.e. newly diagnosed) with AoHD and JoHD compared with matched controls from the general population; (2) understand mortality (timing and cause) in people with JoHD and AoHD; and (3) assess the disease burden (chronic comorbidities, hospitalizations and death) among individuals with an HD diagnosis who were living in Sweden in 2018.

## Methods

### Study design and data source

This was a retrospective, longitudinal cohort study using data from the Swedish National Patient Registry [23] (NPR; coverage start dates: 1987 [inpatient] and 2001 [outpatient]), Prescription Drug Registry [24] (PDR; coverage start date 2005) and Cause of Death Registry [25] (CDR; coverage start date: 1961). Briefly, medical data from the Swedish NPR include main diagnoses, up to 21 secondary diagnoses, and up to 30 surgical procedures from public and private service providers. Diagnoses are coded using the International Classification of Diseases-10 (ICD-10) and surgical or medical procedures with the Nordic Classification of Surgical/Medical Procedures. Primary care is not covered in the Swedish NPR. Prior to receipt, data from the registries were linked through a personal identification number, a unique number issued to all Swedish citizens, offering lifelong, nationwide follow-up. Ethical approval was obtained from the Swedish Ethical review board (Etikprövningsmyndigheten [26]; approval number 2020-07037). The high quality of these databases provides an opportunity to increase our knowledge of the clinical burden of HD, on a national scale and in individuals of all ages.

The study period was between 1 January 2002 and 31 December 2019, whereby individuals with an index date (i.e. approximate date of HD diagnosis) between 2002 and 2018 were included in the study cohort. Cohort enrollment ended 1 year before the end of the follow-up to allow patients to accrue up to 1 year of follow-up after the index.

### Population

#### Incident HD cohort and matched controls from the general population

For the incident cohort, the HD diagnosis date was defined as the first HD healthcare encounter (inpatient or outpatient [at primary or secondary position]) in the individual’s record as reported by a physician in a specialist setting during the cohort enrollment period (2002–2018) (ICD-10-SE: G10–Huntington’s disease [Online Resource 1]). The date of the first code approximates the date of HD diagnosis and marks the start of the individual’s follow-up in the cohort. No age restrictions were applied. It is not possible to reliably assess whether an individual had a genetic test to confirm the HD diagnosis using these data sources. The baseline period was defined as ≥ 1 year prior to a person’s index date, during which baseline characteristics were assessed. Follow-up was defined as the period between the day after the index until death, emigration or the end of the data (whichever came first).

The source population for the matched non-HD general population control group included individuals in the PDR, CDR and medical birth register, a database recording all births in Sweden since 1973 [27]. Non-HD controls were matched to the incident cohort using exact matching on two variables: age (year of birth) and gender; each case was matched to up to four controls. For ease of reference, we will refer to this cohort as matched controls.

#### Prevalent HD cohort

In the prevalent cohort (i.e. individuals who had HD in 2018), people with a relevant diagnostic code (ICD-7: 355.2; ICD-8: 331; ICD-9: 333.4 or 333E; ICD-10: G10 or F02.2, primary or secondary position) in an inpatient or outpatient setting between 1 January 1964 and 1 January 2018 and who were alive on 1 January 2018 (index date) were included in the prevalent cohort. Healthcare burden was assessed in the 1 year (365 days) after the index date (i.e. 1 January 2018 to 31 December 2018). Baseline characteristics were defined as all available data prior to and including 1 January 2018.

### Statistical analysis

#### Patient characteristics

For the incident cohort, demographics at the index for all individuals were summarized and stratified by gender and age, including those aged < 20 years (JoHD cohort) and ≥ 20 years (AoHD cohort).

#### Incidence of clinical conditions and symptomatic therapies

Pre-specified acute and chronic clinical conditions and symptomatic therapies were identified using ICD-10, non-surgical procedure codes or non-medical procedure codes, respectively. The complete code list can be found in Online Resource 1. Either ICD-10 codes for hypertension or Anatomic Therapeutic Chemical (ATC) codes for antihypertensives were used to define hypertension. To measure the clinical burden following an HD diagnosis, IRs (per 100 person-years [PY]) were calculated based on the time from the index to the first clinical event record, without a prior record in all available prior data. This was repeated for each specified acute and chronic clinical event, medication and procedure of interest. Incidence rate ratios (IRRs) were used to describe differences between relative risk/in individuals with HD compared with matched controls. Results were reported by age, stratified into JoHD and AoHD subgroups.

#### Medical history prior to diagnosis

History of Charlson Comorbidity Index scores (CCIs) as well as all specified acute and chronic clinical conditions and therapies of interest, were described by looking back at all available encounters prior to the index (≥ 365 days). The number and proportion (%) of individuals with a history of disease were reported. Results were reported by age, divided into JoHD and AoHD subgroups.

#### Survival and predictors of mortality in HD

The Kaplan–Meier method was used to estimate median time to death and univariable analysis was conducted to assess the association between each baseline covariate and death. Baseline covariates included age at the index, sex, geographic region, CCI, as well as chronic and acute clinical events of interest. Violations of the proportional hazard (PH) assumption were assessed by visual inspection of plots of the Schoenfeld residuals. Only significant covariates were included in the multivariable Cox proportional hazards model to determine the strongest risk factors of death from the list of potential comorbidities, taking into account those variables which are anticipated to be of greatest clinical importance. The top 10 most common underlying causes of death were also described according to ICD-10 codes derived from the CDR database.

#### Clinical characteristics, hospitalization rates and medical history in individuals with HD alive in 2018

The prevalence of HD was calculated as the number of people alive in 2018 with a diagnosis of HD (as described above), divided by the total 2018 Swedish population (*N* = 10,230,185; data source: Statistics Sweden). Patient demographic characteristics as well as comorbidities and history of clinical events were described in the prevalent cohort at baseline. All-cause hospitalization rates (overall, planned and unplanned) were defined as the total number of hospitalizations (inpatient or specialist outpatient including emergency room visits) that occurred in 2018 and described per 100 PY. These were stratified according to the ‘PVARD’ variable in the Swedish NPR, a binary variable that indicates whether a hospital admission was planned or not. The proportion of people (*n*, %) with ≥ 1 record of a cause-specific hospital visit or treatment in all prior medical records was also described. Results were stratified by years since the first HD diagnosis (< 3, 3–5, 6–9 and ≥ 10 years) to better understand how the clinical burden varies throughout the course of the disease.

## Results

### Baseline demographics and characteristics of the incident HD cohort

There were 1492 incident individuals with HD in Sweden from 2002 to 2018, of which 1447 were in the AoHD cohort and 45 were in the JoHD cohort (Table 1). Median (interquartile range [IQR]) age at the index date was 57 (43–68) years and 8 (5–17) years and the proportion of males was 50.1% and 48.9% in the AoHD and JoHD cohorts, respectively (Table 1). Prior to diagnosis (baseline), the majority (> 70%) of individuals had a low comorbidity burden (CCI = 0; Table 1). Individual comorbidities in the CCI can be seen in Table 1 of Online Resource 2.

**Table 1:** Baseline clinical characteristics and comorbidities in the incident AoHD and JoHD cohorts and matched controls

Measure	Incident (AoHD) *N* = 1447	Matched control (AoHD) *N* = 5772	Incident (JoHD) *N* = 45	Matched control (JoHD) *N* = 174
Mean (SD) individual baseline period duration, years (SD)	9.4 (5.0)	9.3 (5.0)	7.0 (4.2)	7.6 (4.4)
Mean individual follow-up period duration, years (SD)	6.9 (4.6)	8.5 (5.0)	10.7 (4.7)	11.0 (4.6)
Median age at the index, years (IQR)	57 (43–68)	57 (43–69)	8 (5–17)	8 (5–16)
Mean age at the index, years (SD)	55.9 (16.7)	56.0 (16.6)	9.5 (6.2)	9.4 (6.0)
Age at the index, categorized, *n* (%)				
< 20 years	0 (0)	0 (0)	45 (100)	174 (100)
20–29 years	88 (6.1)	341 (5.9)	0 (0)	0 (0)
30–39 years	197 (13.6)	797 (13.8)	0 (0)	0 (0)
40–49 years	244 (16.9)	936 (16.2)	0 (0)	0 (0)
50–59 years	282 (19.5)	1143 (19.8)	0 (0)	0 (0)
60–69 years	307 (21.2)	1222 (21.2)	0 (0)	0 (0)
70+ years	329 (22.7)	1333 (23.1)	0 (0)	0 (0)
Gender at the index, *n* (%)				
Male	725 (50.1)	2885 (50.0)	22 (48.9)	85 (48.9)
Female	722 (49.9)	2887 (50.0)	23 (51.1)	89 (51.1)
CCI, categorized, *n* (%)				
0	1069 (73.9)	4902 (84.9)	35 (77.8)	163 (93.7)
≥ 1	378 (26.1)	870 (15.1)	10 (22.2)	11 (6.3)
Acute events (pre-index), *n* (%)				
Neuritis	6 (0.4)	27 (0.5)	0 (0)	0 (0)
Radiculitis	< 5	< 5	0 (0)	0 (0)
Meningitis	8 (0.6)	5 (0.1)	8 (17.8)	0 (0)
Acute psychotic episode	17 (1.2)	12 (0.2)	< 5	0 (0)
Cerebrovascular illness	63 (4.4)	153 (2.7)	0 (0)	0 (0)
Cardiovascular disease (primary diagnosis only)	57 (3.9)	195 (3.4)	< 5	0 (0)
Pneumonia	105 (7.3)	161 (2.8)	< 5	7 (4.0)
Acute respiratory symptoms	226 (15.6)	722 (12.5)	10 (22.2)	14 (8.0)
Fractures	263 (18.2)	610 (10.6)	< 5	10 (5.7)
Subdural hematoma	< 5	5 (0.1)	0 (0)	0 (0)
Bleeding events	183 (12.6)	575 (10.0)	< 5	< 5
Thrombocytopenia	< 5	9 (0.2)	0 (0)	< 5
Constipation	264 (18.2)	811 (14.1)	6 (13.3)	< 5
Chronic events (pre-index), *n* (%)				
Depression	163 (11.3)	173 (3.0)	0 (0)	< 5
Anxiety disorders	113 (7.8)	141 (2.4)	< 5	< 5
Obsessive–compulsive disorder	10 (0.7)	7 (0.1)	< 5	0 (0)
Dementia	149 (10.3)	53 (0.9)	0 (0)	0 (0)
Hydrocephalus	9 (0.6)	6 (0.1)	0 (0)	0 (0)
Epilepsy	63 (4.4)	42 (0.7)	12 (26.7)	0 (0)
Communication and speech problems	19 (1.3)	10 (0.2)	< 5	0 (0)
Hypertension	320 (22.1)	1422 (24.6)	< 5	0 (0)
Chronic renal impairment	25 (1.7)	34 (0.6)	0 (0)	0 (0)
Asthma	53 (3.7)	132 (2.3)	8 (17.8)	8 (4.6)
Liver failure/hepatic impairment/hepatitis	13 (0.9)	29 (0.5)	0 (0)	0 (0)
Gastrointestinal events	341 (23.6)	1146 (19.9)	8 (17.8)	7 (4.0)
Thyroid disorders (hyper or hypothyroid)	50 (3.5)	134 (2.3)	< 5	0 (0)
Bone marrow disorders	52 (3.6)	92 (1.6)	0 (0)	0 (0)
Cancer	107 (7.4)	412 (7.1)	0 (0)	< 5
Antipsychotic use	205 (14.2)	127 (2.2)	< 5	0 (0)
Tetrabenazine use	14 (1.0)	0 (0)	0 (0)	0 (0)
Antidepressants and mood stabilizers	556 (38.4)	1115 (19.3)	6 (13.3)	< 5
Antiepileptic medications	144 (10.0)	191 (3.3)	10 (22.2)	0 (0)
Dopaminergic agents	69 (4.8)	68 (1.2)	0 (0)	0 (0)
PEG feeding	9 (0.6)	7 (0.1)	< 5	0 (0)

Due to Swedish confidentiality laws, categories observed in < 5 people are marked as *n* < 5

*AoHD* adult-onset Huntington’s disease, *CCI* Charlson Comorbidity Index, *IQR* interquartile range, *JoHD* juvenile-onset HD, *PEG* percutaneous endoscopic gastrostomy, *SD* standard deviation

### Medical history prior to diagnosis

For both the AoHD and JoHD cohorts, the history of acute/chronic clinical events and therapies at baseline were consistently higher in individuals with HD than people in matched controls.

For acute events, the greatest proportional differences in the AoHD cohort between cases and matched controls were observed for pneumonia (7.3 vs. 2.8%), constipation (18.2 vs. 14.1%), fractures (18.2 vs 10.6%) and acute respiratory symptoms (15.6 vs. 12.5%; Table 1), respectively. For chronic events, the biggest differences were observed for dementia (10.3 vs. 0.9%), depression (11.3 vs. 3.0%) and anxiety disorders (7.8 vs. 2.4%). Hypertension was slightly more common in matched controls (24.6 vs. 22.1%). The AoHD cohort had a greater history of all symptomatic therapies, of which antipsychotics (14.2 vs 2.2%), antidepressants and mood stabilizers (38.4 vs. 19.3%) and antiepileptic medications (10 vs. 3%) showed the biggest difference compared with matched controls. Only 1% were on tetrabenazine prior to diagnosis.

In the JoHD cohort, the biggest differences between cases and matched controls for acute events were observed in the prevalence of meningitis (17.8 vs. 0%), respiratory symptoms (22.2 vs. 8.0%) and constipation (13.3 vs. 1.7%). For chronic events, epilepsy (26.7 vs. 0%), gastrointestinal events (17.8 vs. 4.0%) and asthma (17.8 vs. 4.6%) showed the biggest difference compared with matched controls. History of fractures, thrombocytopenia, depression and cancer were marginally higher in matched controls (Table 1). For symptomatic therapies, the greatest difference observed between JoHD cases and matched controls was for antiepileptic medications (22.2 vs. 0%).

### Incidence of clinical conditions and symptomatic therapies

**Fig. 1:**
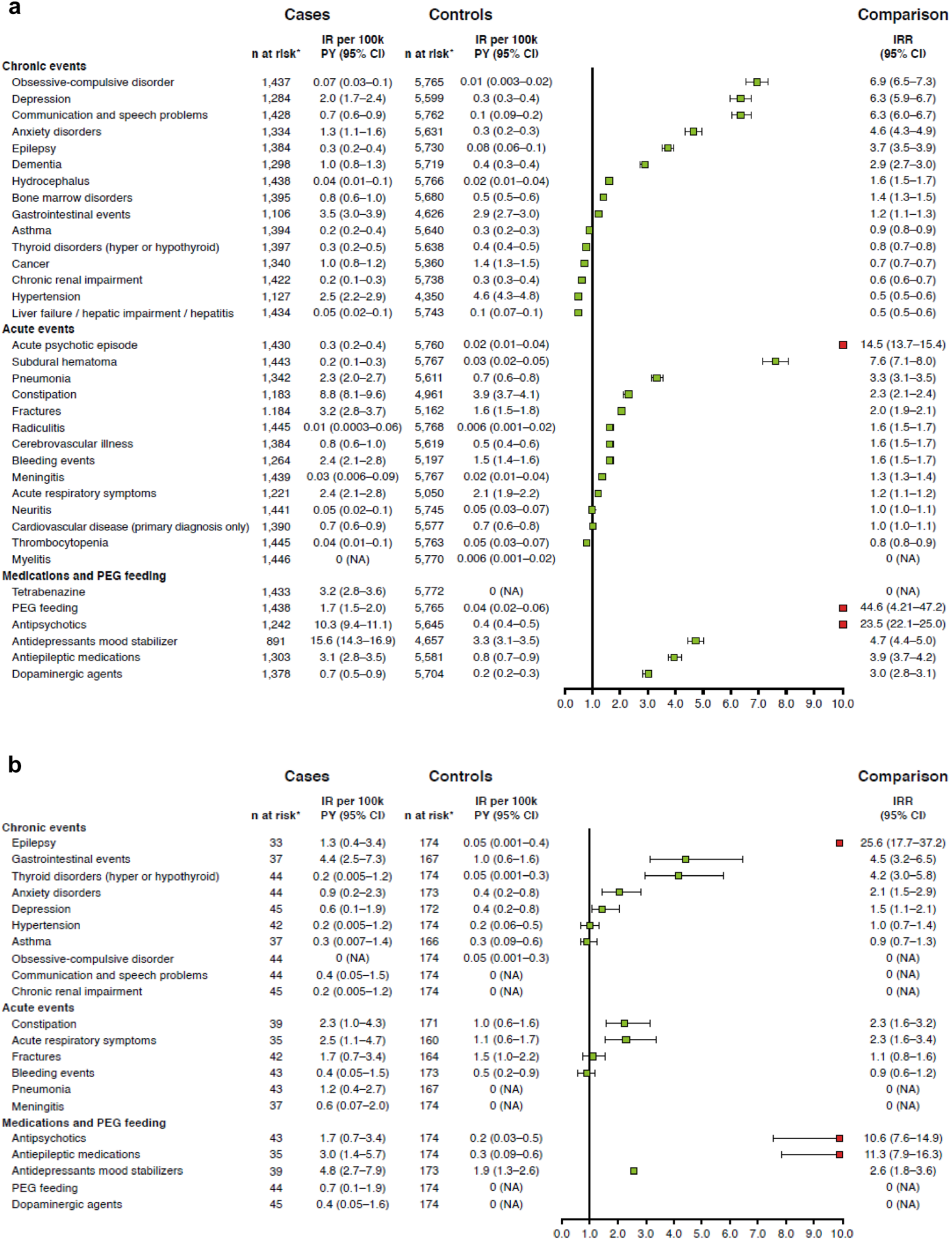
First-event IRs and IRRs of acute/chronic events and symptomatic therapies recorded in **A** the AoHD cohort and **B** the JoHD cohort compared with matched controls. *Categories with < 5 cases in both HD cases and matched control groups have not been included in the plot. Code categories that were excluded in **B** include dementia, hydrocephalus, bone marrow disorders, cancer, liver failure, myelitis, neuritis, radiculitis, acute psychotic episode, cerebrovascular illness, cerebrovascular disease, subdural hematoma, thrombocytopenia, tetrabenazine. *AoHD* adult-onset Huntington’s disease, *CI* confidence interval, *IR* incidence rate, *IRR* incidence rate ratio, *JoHD* juvenile-onset HD, *NA* not applicable; PEG, Percutaneous Endoscopic Gastrostomy; PY, patient-years.

The IR of acute and chronic events and symptomatic therapies was defined as the first record (i.e. new event) after HD diagnosis without a previous record in all prior data. These were generally much higher in people with HD than in matched controls according to the IRRs, in both the AoHD and JoHD cohorts (Fig. 1).

For individuals in the AoHD cohort, the IRR of acute events (95% confidence interval [CI]) was highest for acute psychotic episodes (14.5 [13.7–15.4]), subdural hematoma (SDH) (7.6 [7.1–8.0]) and pneumonia (3.3 [3.1–3.5]). The highest IRRs (95% CI) for chronic events were for obsessive–compulsive disorders (6.9 [6.5–7.3]), communication and speech problems (6.3 [6.0–6.7]) and depression (6.3 [5.9–6.7]). Several events had a higher IRR in the matched control cohort, including thrombocytopenia (0.8 [0.8–0.9]), hypertension (0.5 [0.5–0.6]) and cancer (0.7 [0.7–0.7]; Fig. 1a) Incidence of all symptomatic therapies was higher in the AoHD cohort compared with matched controls. The largest difference was for tetrabenazine (44.6 [4.21–47.2], Percutaneous Endoscopic Gastrostomy (PEG) feeding (23.5 [22.1–25.0] and antipsychotics (4.7 [4.4-5.0]), followed by antidepressants, antiepileptics and dopaminergic agents.

For individuals in the JoHD cohort, acute events with the highest IRRs were acute respiratory symptoms (2.3 [1.6–3.4]) and constipation (2.3 [1.6–3.2]). The highest IRRs for chronic events were for epilepsy (25.6 [17.7–37.2]), gastrointestinal events (4.5 [3.2–6.5]) and thyroid disorders (4.2 [3.0–5.8]). The only acute event with a greater IRR in the matched control cohort was bleeding events (0.9 [0.6–1.2]) (Fig. 1b).

### Mortality

In the AoHD cohort, a total of 563 (38.9%) people died during the total study follow-up period compared with < 5 people in the JoHD cohort. The median time from the index to all-cause mortality was 12.1 years in the AoHD cohort. The 10 most common causes of death in the AoHD cohort were HD (41%), followed by acute myocardial infarction (1.8%), chronic ischemic heart disease (1.5%), unspecified dementia (1.5%), pneumonia (1.4%), malignant neoplasm of unspecified part of bronchus or lung (1.4%), Parkinson’s disease (1.4%), frontotemporal dementia (1.4%), atherosclerotic heart disease of native coronary artery (1.4%) and cerebral infarction (1.4%). In the JoHD cohort, the most common cause of death was also HD. The low number of JoHD deaths prevented the estimation of median survival time in this cohort, and deaths were, therefore, pooled across all patients for the Cox analyses. Univariable-identified age; gender; region; CCI score; dementia; communication and speech problems; hypertension; chronic renal impairment; gastrointestinal events; thyroid disorders; bone marrow disorders; cancer; antipsychotics; antidepressants and mood stabilizers; antiepileptic medication; dopaminergic agent use; radiculitis; cerebrovascular disease; pneumonia; acute respiratory symptoms; constipation; factures; and bleeding events were associated with an increased risk of death. After adjustment for these covariates in the multivariable model, only age (≥ 40 years), female sex, CCI score and pneumonia were independently associated with the risk of death (Table 2 of Online Resource 2).

### Clinical characteristics, hospitalization rates and medical history in individuals with HD in 2018

A total of 1039 people were living with a diagnosis of HD in Sweden in 2018. The prevalence of HD in Sweden in 2018 was, therefore, approximately 10.2 per 100,000. The median age (IQR) was 57 (45–70) years, of which 53.7% were female (Table 3 of Online Resource 2). The median age at diagnosis was 50 (38–62) years, and the most common age strata at diagnosis was 40–49 years (*N* = 203, 19.5%). Only 27 individuals (2.6%) were living with a diagnosis of HD under the age of 20 years. In 4% of HD cases, the HD diagnosis date was unknown (those with a diagnosis before data availability in 2001) and these HD cases were, therefore, categorized into the ≥ 10 years since onset subgroup. While age on 1 January 2018 was similar across subgroups, age at diagnosis was lower in those diagnosed ≥ 10 years ago (46; IQR 34–55 years) compared with those diagnosed < 3 years ago (57; IQR 42–72 years). Demographics broken down by years since onset can be found in Table 3 of Online Resource 2.

In 2018, the all-cause hospitalization rate was 50.1 per 100 PY (95% CI 45.9–54.6). This was higher the ≥ 10 years since onset subgroup (59.8 [51.8–68.7]) compared with the < 3 years from onset subgroup (42.3 [34.4–51.3]) per 100 PY. Hospitalizations that were not planned were more common than planned hospitalizations (42.3 [95% CI 38.5–46.5] vs. 7.8 [95% CI 6.2–9.7] per 100 PY), and this difference was more pronounced in the ≥ 10 years since onset subgroup (55.4 vs. 4.5 events per 100 PY) compared with the < 3 years from onset subgroup (31.0 vs. 11.3 events per 100 PY). Of a total 58 deaths that occurred in people with HD during 2018, HD was reported as the main cause for 28 (48.3%).

**Fig. 2:**
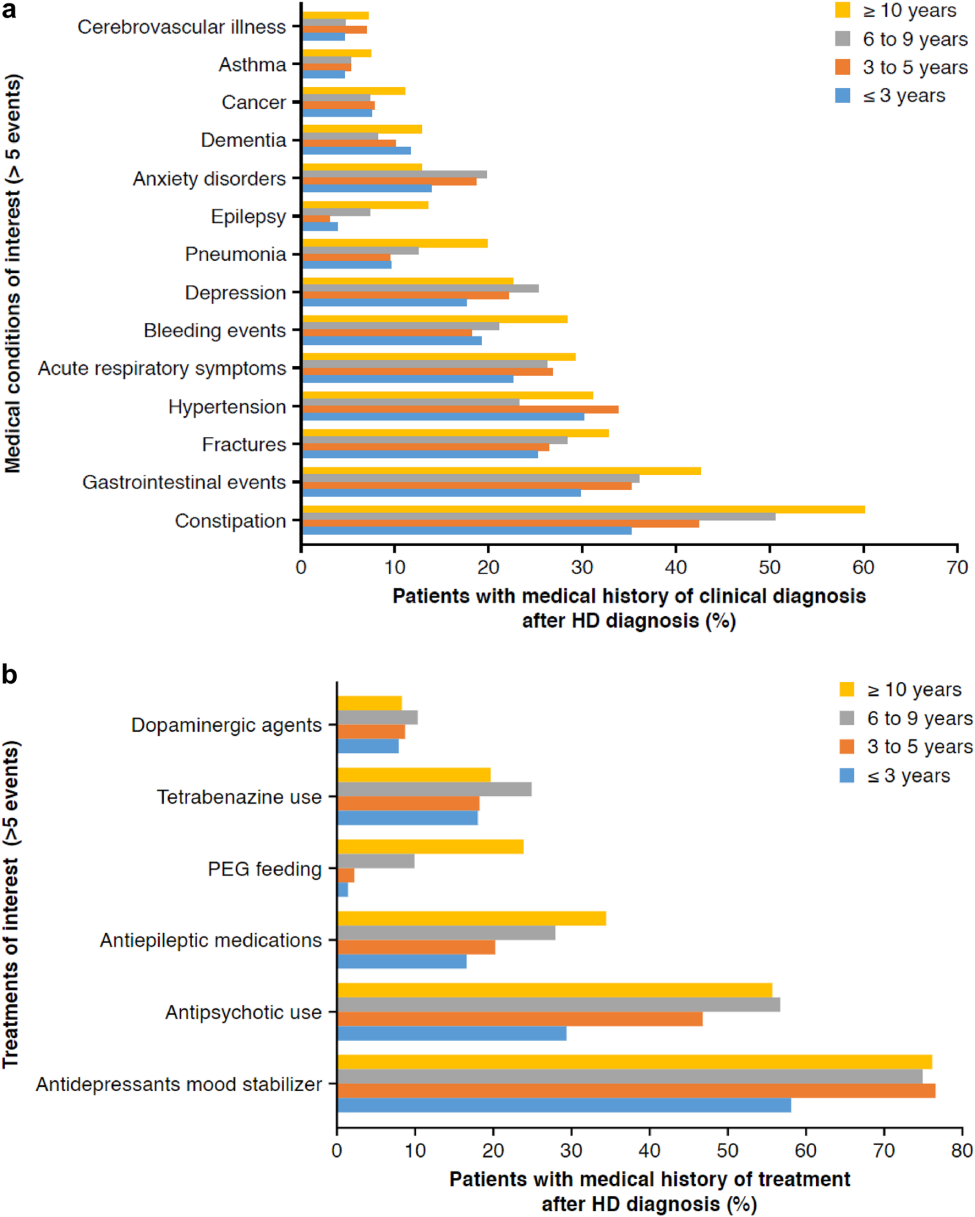
Medical history of clinical conditions **A** and treatments **B** in people with HD alive on 1 January 2018, stratified by years since the first HD diagnosis

A pattern of higher clinical burden with years since diagnosis was apparent, whereby more people in the ≥ 10 years since diagnosis subgroup had a medical history of nearly all studied clinical diagnoses and treatments, compared with those in the < 3 years since diagnosis subgroup (Fig. 2a). Only anxiety (13.8 vs. 12.8%), cardiovascular disease (5.0 vs. 4.5%) and bone marrow disorders (6.3 vs. 3.0%) were higher in people in the < 3 years since diagnosis subgroup. Median (IQR) age (56–58 years) and look-back duration (17 years) were similar across all subgroups (Table 3 of Online Resource 2).

History of PEG feeding was increasingly more common in those who had lived with an HD diagnosis for longer (*n* < 5, 2.2%, 9.9% and 23.8% for < 3, 3–5, 6–9 and ≥ 10-year categories respectively). Antidepressants were the most common symptomatic therapy, whereby 58–76% of individuals had a prior history of antidepressants at some point prior to 2018 (Fig. 2b).

## Discussion

The present study describes the clinical burden of HD in a cohort of 1492 incident patients with HD, including 45 patients with JoHD, over a 17-year period by utilizing the nationwide Swedish registries between 2002 and 2019. The nationwide coverage and comprehensive linkage of these secondary care records make this one of the most extensive longitudinal cohort studies of individuals with HD, and the largest population-based study to assess the clinical burden in JoHD to date.

The Swedish national registries were selected for use in this analysis as they contain longitudinal patient data covering the whole nation of Sweden (i.e. 99% of the population), thus providing a valuable insight into the burden of AoHD and JoHD, without the risk of selection bias inherent in other cohort studies. Swedish national registries allow for a very long follow-up period, as individuals can only drop out of the registries in the event of emigration or death. The registries undergo rigorous quality checking before being released [28] and, therefore, are considered to be high-quality sources of data [29]. However, although these data have advantages over survey data collected prospectively, the data might be somewhat limited since some clinical variables, e.g. premanifest versus motor-manifest, are lacking.

The results described here may be useful for clinicians to understand the elevated risk of relevant clinical outcomes in individuals with HD compared with a cohort of matched controls from the general population. This comparison is particularly useful for estimating the incidence of rare events that cannot be detected in smaller sample sizes due to issues with power. IRs of important clinical outcomes in the years following a first diagnosis could be used to guide treatment planning and educate patients on the natural history of HD. Our results also provide a cross-sectional snapshot of disease burden, by describing the distribution of demographics and clinical characteristics of patients living with HD in 2018. Taken together, these findings increase our understanding of the physical, psychiatric and healthcare burden of HD in Sweden, which can reasonably be assumed to reflect the disease burden across other countries in Western Europe with similar healthcare systems.

One of the most apparent findings of this study was the psychiatric burden experienced by patients, both before and after their first HD diagnosis. During follow-up, people with AoHD had a higher incidence of developing obsessive–compulsive disorders, acute psychotic episodes, communication/speech problems, depression, anxiety and dementia compared with matched controls. Even prior to diagnosis, these psychiatric signs were more than twice as common in people with AoHD than in matched controls, corroborating previous research that psychiatric symptoms typically precede a clinical diagnosis (which is largely determined by the presence of motor symptoms and confirmatory genetic testing) [30]. In addition to the well-reported early cognitive and psychiatric comorbidities [18, 30], our results also reflect the progressive loss of speech, whereby ~ 22% of patients with AoHD already had a record of communication or speech disturbance before their HD diagnosis [31]. Psychiatric and cognitive decline places a huge burden on patients and their families, and our prodromal findings emphasize the need for careful monitoring of at-risk HD gene carriers and appropriate treatment planning.

Beyond the psychiatric burden of HD, we add to the literature on other acute clinical outcomes in the AoHD population, including a higher incidence of SDH, pneumonia, constipation and fractures. SDH is common in people with HD because of inherent features such as cortical brain atrophy, balance impairment, and head trauma due to a high frequency of falls [32, 33]. Here, we identified 34 adult cases (2.3%) of SDH (0 juvenile cases), which is well aligned with a recent chart review conducted at specialist HD centers in the USA which estimated that ~ 2% of manifest patients experience SDH. Conversely, we estimated a lower incidence of SDH compared with a Finnish nationwide cohort study of 192 patients with HD [34] (0.2 vs. 470 per 100,000 PY), which may be attributed to their smaller cohort size. Whilst our study was not designed to explore the causal risks for SDH, we also noted a higher risk of fractures in HD compared with matched controls, which may be linked to loss of balance and the increased falls described, and which are thought to be precursors to SDH. Perhaps less well described in the literature, we found that patients with AoHD had double the rate of constipation compared with matched controls in the years after diagnosis. It is likely that constipation is still under-reported or under-diagnosed in our study since other symptoms may be more prominent, and it is not clear whether this is attributable to the gastrointestinal problems associated with progressive HD, or whether this could be a side effect of the medications patients are receiving [35]. Nonetheless, constipation could exert substantial discomfort and indirectly impact the quality of life and may be worthy of consideration.

We observed a lower incidence rate of cancer in the HD population compared with the general population, in line with several earlier studies. A Danish study of 694 individuals with HD covering the period 1943–1993 reported a lower incidence of cancer in all major tissues except the buccal cavity and the pharynx compared with national incidence rates for various categories of tumors [36]. Another study based on Swedish registries from 1969–2008 also reported lower cancer incidence for the included 1510 individuals with HD, as well as for individuals with other polyglutamine diseases [37]. Additionally, a decreased observed cancer incidence was reported for 372 individuals with HD compared with expected cases [38]. Finally, reduced cancer incidence was also reported for the 6540 HD gene carriers included in the observational European REGISTRY study [39]. Interestingly, the HD-causing protein huntingtin, including its wild-type form, has been suggested to regulate cellular processes involved in tumorigenesis and cancer progression [40]. Hence, there may be a link between HD and cancer, although the underlying mechanisms are likely complex and not yet fully understood. It is also important to consider that results of a lower incidence rate of cancer in the HD population may be influenced by reporting bias, as individuals with HD may not be able to communicate oncogenic symptoms to their clinicians, leading to an underestimation of cancer in routine clinical settings.

In addition to clinical diagnoses, we were also interested in assessing the incidence of common symptomatic treatments. Consistent with the finding of increased psychiatric burden in other literature [18], we found a higher incidence of all psychiatric medication use in the AoHD cohort compared with the matched control cohort. The relative incidence of antipsychotic prescriptions showed the biggest difference between AoHD and matched control cohorts. Whilst this finding was expected, it is important to note that we cannot rule out the use of some antipsychotics (such as haloperidol, risperidone, olanzapine and aripiprazole) for the treatment of involuntary movements, and not just psychosis [41]. Nonetheless, the higher use of these psychiatric medications in the AoHD cohort highlights the high burden that HD exerts on healthcare systems worldwide and the unmet need for disease-modifying therapies or other psychological therapies to treat these symptoms early. Beyond psychiatric medications, we also looked at the prescription of tetrabenazine, a monoamine inhibitor that is currently the only treatment indicated specifically for the treatment of motor abnormalities in HD in Sweden. Only 1% of patients had been prescribed tetrabenazine in the years before diagnosis, which rose to 19% in the years after diagnosis; this was low compared with the incidence of tetrabenazine use in a recent retrospective study in Israel (63%) [42]. Finally, we also explored the incidence of PEG feeding, a means of bypassing the oral cavity to provide hydration and nutrition directly into the gastrointestinal system, with the hope of reducing complications including pneumonia, malnutrition and immobility. Whilst PEG feeding appears to increase life expectancy, there is ongoing discussion around the impact on patient quality of life [43]. In our cohort, 11% of incident patients with AoHD (and < 5 patients with JoHD) had a record of PEG feeding during follow-up; however, it is possible that this may be underestimated since not all patients in our study would have reached advanced stages of HD during the follow-up period.

A strength of this study is the comparatively large juvenile cohort relative to previous studies. Compared with matched controls, patients with JoHD had a higher incidence of epilepsy, gastrointestinal events, thyroid disorders, acute respiratory symptoms and constipation in the time following diagnosis. The most striking difference was in the rate of epilepsy which was ~ 26 times greater than the matched control cohort. Other studies have described the presentation of seizures in JoHD [10, 44], but to our knowledge, this is the only population study to investigate the occurrence of epilepsy using administrative clinical records. A previous retrospective chart review study estimated that ~ 38% of patients with JoHD presented with seizures during the course of the disease [11] and a more recent systematic review estimated that ~ 15% of patients with JoHD have seizures or epilepsy at presentation [10]. We observed that 16/45 (36%) people with JoHD had a record of epilepsy at some time before or after diagnosis, which is very similar to the estimate by Cloud et al. [11]. When we indirectly compared with the AoHD cohort, those with JoHD were more likely to receive a diagnosis of epilepsy, gastrointestinal events and acute respiratory symptoms in the years after diagnosis. Regarding epilepsy, history of epilepsy prior to HD diagnosis was 6 times greater in JoHD compared with AoHD and incidence risk after diagnosis was also higher in JoHD (IRR 25.6 vs. 3.7). It has been suggested previously that elevated epilepsy risk is unique to JoHD and occurrence in AoHD is similar to the general population [13]; however, our findings suggest that, whilst lower than the JoHD group, epilepsy does seem to be a sign in AoHD also. Increased incidence of thyroid disorders compared with matched controls was an intriguing finding due to the suggestion that loss of neurons in the hypothalamus could lead to neuroendocrine disruption [45] and may be implicated in the non-motor symptoms of HD [46]. Nonetheless, caution is encouraged when interpreting these numbers due to the small number of JoHD cases. The challenges around diagnosing JoHD have been discussed previously [13], in part due to the domination of cognitive and behavioral problems. Our results show that meningitis, constipation, epilepsy, hypertension and asthma are higher in JoHD prior to an HD diagnosis than in matched controls. This sheds light on some of the prodromal signs and symptoms of HD, which could be considered by clinicians during the clinical work-up prior to making a clinical diagnosis of JoHD.

The long follow-up time and complete death recording make this an ideal study to assess mortality in patients with a diagnosis of HD. The median survival time in the AoHD cohort was 12.1 years, which is similar to recent population-based cohort studies of HD in Israel (12 years [42]) and the UK (12.4 years [47]), which also define the index as the first diagnosis in the patient’s medical records and take place during a similar time window. Other studies have reported median survival up to 25 years [48–50] which may be attributable to the index definition, study methods and accuracy of death reporting. Better understanding of survival time will be important for epidemiologists who wish to model the long-term effects of disease-modifying therapies in the future, which will hopefully extend and improve life for those with HD. Our findings around the cause of death align with previous studies [48–51] (Furby H, et al. Unpublished observations with written permission from the source) in which HD was recorded as the main underlying cause of death. Often patients develop intercurrent infections leading to death, and this knowledge can help with end-of-life planning. For instance, pneumonia is a common complication in the advanced stages of HD, as patients have difficulty swallowing, which in turn can lead to choking and aspiration pneumonia [52]. Multiple studies have reported pneumonia as the leading intermediate cause of death in HD [49, 50, 52], a finding that is indirectly supported by our multivariate analyses which revealed that pneumonia was a significant risk factor for predicting all-cause mortality (hazard ratio 2.16). Anecdotal evidence exists for higher levels of smoking in individuals with HD compared with matched controls; however, since smoking history was not collected in this study, we are unable to rule out whether higher levels of pneumonia in people with HD may be partially attributed to the effects of smoking.

A novel aspect of our study comes from the cross-sectional analysis, which shed light on the demographics and clinical burden of those living with HD in Sweden in 2018. We ascertained that 1039 people (all ages) were living with an HD diagnosis in Sweden in 2018, which equates to 10.2 per 100,000 people living in Sweden in 2018. This is in line with other prevalence estimates across North America, north-western Europe and Australia (5.96–13.7 per 100,000) [53]. Our results imply a pattern of increasing clinical burden associated with the number of years since diagnosis. The all-cause hospitalization rate was greater in the ≥ 10 years since HD onset subgroup when compared with the < 3 years since onset subgroup, with unplanned hospitalizations being more common. This demonstrates the increasing burden on healthcare systems over the natural history of disease. History of all diagnoses and treatments studied, except anxiety and bone marrow disorders, were higher in the ≥ 10 years since onset subgroup compared with the < 3 years since onset subgroup. It is unlikely that this was attributable to natural aging since age was similar between subgroups. In the absence of HD-specific clinical outcomes to determine disease stage, years from diagnosis is only a proxy; however, the increasing prevalence of PEG feeding with time since diagnosis helps to support this conclusion, since PEG feeding is only used to treat those in the most advanced stages of HD who can no longer self-feed. We believe that this cross-sectional snapshot may help healthcare planners and public health experts to better understand the clinical profile of people with HD, and the relevant healthcare demands that HD places on healthcare systems.

Due to the long follow-up (median 17 years) of patients in this study, this data source enables us to report on rare events with greater sensitivity. In contrast to other observational studies such as academic or disease-specific registry studies from isolated centers, this database captures all patients with HD in Sweden since 2001 and as such is not subject to the selection bias that is inherent in many studies conducted in specialist HD centers, whereby patients may receive an earlier diagnosis, better disease management and, possibly, better clinical outcomes.

### Limitations

Clinical measures specific to HD such as disease stage, motor symptoms or CAG repeat length are typically not captured in a routine healthcare setting, and so we could not include these in our analyses. For example, CAG expansion is known to predict faster progression and earlier mortality [54]. Similarly, without an exact date of clinical diagnosis, we used the date of the first specialist inpatient or secondary care record as a proxy for diagnosis. Those at risk of HD may often avoid a formal diagnosis until they are emotionally ready, or until the clinician is confident in the diagnosis, which may explain why the age of index is older than the 30- to 50-year age range commonly quoted in the literature [44]. We cannot completely rule out misclassification of premanifest patients as manifest (i.e. if they received a positive genetic text) or that the patient had been motor manifest for some time prior to secondary care diagnosis. Indeed, there were a few cases of tetrabenazine use prior to the index, suggesting that they were already motor manifest at diagnosis. It is not possible to rule out whether the antiepileptic medications assessed in this analysis, such as lamotrigine or valproic acid, were prescribed for the treatment of epilepsy or as mood stabilizers. The rare nature of JoHD meant that we were unable to report all clinical outcomes in this group, either due to patient anonymity or for statistical robustness. Another common caveat of medical records is the reliance on physicians to accurately and completely record data. People with HD are often under close management by a clinician and we cannot rule out a surveillance bias in this study, where other conditions may be over- or under-reported due to the frequency in which they are seen.

## Conclusions

Our results will help healthcare providers, epidemiologists, policymakers and drug developers to contextualize the background rate of comorbidity events between large case and matched control populations by providing recent estimates on the incidence of acute events, chronic events and mortality in HD. Our findings provide an in-depth description of the natural history of AoHD and JoHD, prior to and in the years after diagnosis, and a clinical summary of patients living with HD in 2018.

## Supplementary Information

Below is the link to the electronic supplementary material.

Supplementary file1 (DOCX 48 KB)

Supplementary file2 (DOCX 213 KB)

## Data Availability

Data are available from Hannah Furby with the permission of the Swedish National Patient Registry, Prescription Drug Registry, Cause of Death Registry and Medical Birth Register.
