# Supplementary material for: Comorbidities and clinical outcomes in adult- and juvenile-onset Huntington’s disease: a study of linked Swedish National Registries (2002–2019)
Source: J Neurol. 2022 Oct 18;270(2):864–76. doi: 10.1007/s00415-022-11418-y (PMC9886595; doi:10.1007/s00415-022-11418-y)
Supplement: Supplementary file 1 — Supplementary file1 (DOCX 48 KB) [file 415_2022_11418_MOESM1_ESM.docx]

**Comorbidities and clinical outcomes in adult- and juvenile-onset Huntington’s disease: A study of linked Swedish National Registries (2002–2019)**

Hannah Furby,^1^ Suzanne Moore,^2^ Anna-Lena Nordstroem,^2^ Richard Houghton,^2^ Dimitra Lambrelli,^3^ Sophie Graham,^3^ Per Svenningsson,^4^ Åsa Petersén^5^

(1) Roche Products Ltd, Welwyn Garden City, UK

(2) F. Hoffmann-La Roche Ltd, Basel, Switzerland

(3) Evidera, London, UK

(4) Karolinska Institutet, Stockholm, Sweden

(5) Translational Neuroendocrine Research Unit, Department of Experimental Medical Science, Medical Faculty, Lund University, Lund, Sweden

**Online Resource 2**

**Supplementary Table 1: Pre-index comorbidities**

| **Comorbidities in the CCI (pre-index), n (%)** | Incident  (AoHD)  N=1,447 | Control  (AoHD) N=5,772 | Incident  (JoHD) N=45 | Control  (JoHD) N=174 |
| --- | --- | --- | --- | --- |
| Myocardial Infarction | 35 (2.4) | 138 (2.4) | 0 (0) | 0 (0) |
| Dementia | 149 (10.3) | 53 (0.9) | 0 (0) | 0 (0) |
| Congestive Heart Failure | 65 (4.5) | 147 (2.5) | n<5 | 0 (0) |
| Peripheral Vascular Disease | 30 (2.1) | 95 (1.6) | 0 (0) | 0 (0) |
| Cerebrovascular Disease | 99 (6.8) | 227 (3.9) | 0 (0) | 0 (0) |
| Chronic Pulmonary Disease | 85 (5.9) | 216 (3.7) | 8 (17.8) | 8 (4.6) |
| Peptic Ulcer Disease | 28 (1.9) | 71 (1.2) | 0 (0) | 0 (0) |
| Rheumatic Disease | 29 (2.0) | 115 (2.0) | 0 (0) | n<5 |
| Diabetes (no chronic complications) | 108 (7.5) | 238 (4.1) | 5 (11.1) | 0 (0) |
| Diabetes (with chronic complications) | 58 (4.0) | 127 (2.2) | n<5 | 0 (0) |
| Mild Liver Disease | 15 (1.0) | 43 (0.7) | 0 (0) | n<5 |
| Moderate or Severe Liver Disease | n<5 | 7 (0.1) | 0 (0) | 0 (0) |
| Renal Disease | 39 (2.7) | 51 (0.9) | 0 (0) | 0 (0) |
| Any Malignancy | 82 (5.7) | 302 (5.2) | 0 (0) | n<5 |
| Metastatic Solid Tumor | 12 (0.8) | 36 (0.6) | 0 (0) | 0 (0) |
| Hemiplegia or Paraplegia | 18 (1.2) | 13 (0.2) | 0 (0) | 0 (0) |

Due to Swedish confidentiality laws, categories observed in <5 people are marked as n<5.

AoHD=adult-onset Huntington’s disease; CCI=Charlson Comorbidity Index; JoHD=juvenile-onset Huntington’s disease.

**Supplementary Table 2: Multivariable Cox regressions: predictors for mortality in the incident HD cohort**

|  | **Individuals with HD** | | **Cox regression** | | |
| --- | --- | --- | --- | --- | --- |
|  | Total | Events | Hazard ratio | Lower 95% CI | Upper 95% CI |
| **Total** | 1,492 | 566 |  |  |  |
| **Age at index (categorized)** |  |  |  |  |  |
| <20 years | 45 | n<5 | Ref |  |  |
| 20–29 years | 88 | 9 | 2.28 | 0.616 | 8.443 |
| 30–39 years | 197 | 24 | 2.432 | 0.73 | 8.099 |
| 40–49 years | 244 | 58 | 5.327 | 1.663 | 17.065 |
| 50–59 years | 282 | 102 | 8.407 | 2.656 | 26.609 |
| 60–69 years | 307 | 156 | 14.201 | 4.51 | 44.721 |
| 70+ years | 329 | 214 | 32.585 | 10.3 | 103.082 |
| **Gender** |  |  |  |  |  |
| Male | 747 | 326 | Ref |  |  |
| Female | 745 | 240 | 0.668 | 0.564 | 0.791 |
| **Geographic region** |  |  |  |  |  |
| East Sweden | 600 | 213 | Ref |  |  |
| South Sweden | 618 | 245 | 1.046 | 0.868 | 1.261 |
| North Sweden | 274 | 108 | 1.063 | 0.84 | 1.344 |
| **Charlson Comorbidity Index group** |  |  |  |  |  |
| 0 | 1104 | 349 | Ref |  |  |
| 1 | 94 | 36 | 1.159 | 0.814 | 1.65 |
| 2 | 185 | 112 | 1.642 | 1.188 | 2.271 |
| 3 | 40 | 25 | 2.021 | 1.228 | 3.326 |
| ≥4 | 69 | 44 | 1.781 | 1.108 | 2.865 |
| **Chronic events prior to baseline** |  |  |  |  |  |
| Dementia | 149 | 91 | 0.813 | 0.588 | 1.126 |
| Hypertension | 323 | 146 | 0.971 | 0.773 | 1.22 |
| Liver failure/hepatic impairment/hepatitis | 13 | 6 | 0.64 | 0.27 | 1.514 |
| Cancer | 107 | 61 | 1.02 | 0.729 | 1.427 |
| **Acute events prior to baseline** |  |  |  |  |  |
| Cardiovascular disease (MI, cardiac arrest or heart failure) | 58 | 36 | 1.031 | 0.678 | 1.566 |
| Pneumonia | 107 | 61 | 2.164 | 1.609 | 2.911 |

Due to Swedish confidentiality laws, categories observed in <5 people are marked as n<5.
CI=confidence interval; HD=Huntington’s disease; MI=myocardial infarction.

**Supplementary Table 3. Demographics and medical history of key clinical diagnoses and treatments in people living with HD in 2018, stratified by years since diagnosis**

|  |  | **Years since diagnosis** | | | |
| --- | --- | --- | --- | --- | --- |
|  | **Overall** | **<3** | **3–5** | **6–9** | ≥**10** |
| **Measure** | **N=1,039** | **N=239** | **N=231** | **N=233** | **N=336** |
| **Age on 1 January 2018** |  |  |  |  |  |
| Mean (SD) | 56.5 (17.2) | 57.2 (18.9) | 56.5 (16.3) | 55.7 (16.8) | 56.5 (16.9) |
| Median (range) | 57 (3–95) | 58 (5–95) | 57 (3–93) | 56 (11–93) | 58 (14–90) |
| Interquartile range | 45–70 | 44–74 | 44–69 | 44–68 | 46–69 |
| **Age at first HD diagnosis** |  |  |  |  |  |
| Mean (SD) | 49.5 (17.7) | 55.7 (19.0) | 52.1 (16.2) | 47.7 (16.8) | 43.7 (16.3) |
| Median (range) | 50 (0-95) | 57 (3–95) | 52 (0–89) | 47 (3–84) | 46 (0–79) |
| Interquartile range | 38-52 | 42–72 | 39–65 | 36–60 | 34–55 |
| **Age at first HD diagnosis, categorized** |  |  |  |  |  |
| <20 years | 45 (4.3%) | n<10^b^ | n<5 | 10 (4.3%) | 25 (7.4%) |
| 20–29 years | 74 (7.1%) | 14 (5.9%) | 17 (7.4%) | 20 (8.6%) | 23 (6.8%) |
| 30–39 years | 173 (16.7%) | 32 (13.4%) | 37 (16.0%) | 46 (19.7%) | 58 (17.3%) |
| 40–49 years | 203 (19.5%) | 41 (17.2%) | 46 (19.9%) | 48 (20.6%) | 68 (20.2%) |
| 50–59 years | 199 (19.2%) | 36 (15.1%) | 46 (19.9%) | 45 (19.3%) | 72 (21.4%) |
| 60–69 years | 170 (16.4%) | 41 (17.2%) | 46 (19.9%) | 44 (18.9%) | 39 (11.6%) |
| >70 years | 133 (12.8%) | 69 (28.9%) | 35 (15.2%) | 20 (8.6%) | 9 (2.7%) |
| **Gender** |  |  |  |  |  |
| Male | 481 (46.3%) | 120 (50.2%) | 104 (45.0%) | 96 (41.2%) | 161 (47.9%) |
| Female | 558 (53.7%) | 119 (49.8%) | 127 (55.0%) | 137 (58.8%) | 175 (52.1%) |
| **Look-back duration (years)** |  |  |  |  |  |
| Mean (SD) | 16.8 (1.5) | 16.4 (2.8) | 16.8 (1.1) | 16.8 (1.1) | 17.0 (0.3) |
| Median (range) | 17 (1–17) | 17 (1–17) | 17 (5–17) | 17 (7–17) | 17 (12–17) |
| Interquartile range | 17–17 | 17–17 | 17–17 | 17–17 | 17–17 |
| **Years since first HD diagnosis** |  |  |  |  |  |
| Mean (SD) | 8.9 (8.1) | 1.4 (0.9) | 4.5 (0.9) | 7.9 (1.1) | 18.0 (8.0) |
| Median (range) | 7 (0–31) | 1 (0–3) | 4 (3–6) | 8 (6–10) | 14 (10–31) |
| Interquartile range | 3–12 | 1–2 | 4–5 | 7–9 | 12–31 |
| **Acute events****^a^** |  |  |  |  |  |
| Myelitis | n<5 | 0 (0%) | 0 (0%) | n<5 | n<5 |
| Neuritis | 8 (0.8%) | n<5 | n<5 | n<5 | n<5 |
| Radiculitis | n<5 | n<5 | n<5 | 0 (0%) | 0 (0%) |
| Meningitis | 18 (1.7%) | n<5 | n<5 | n<5 | 10 (3.0%) |
| Acute psychiatric episode | 34 (3.3%) | 6 (2.5%) | 6 (2.6%) | 8 (3.4%) | 14 (4.2%) |
| Cerebrovascular illness | 62 (6.0%) | 11 (4.6%) | 16 (6.9%) | 11 (4.7%) | 24 (7.1%) |
| Cardiovascular disease (primary diagnosis only) | 48 (4.6%) | 12 (5.0%) | 15 (6.5%) | 6 (2.6%) | 15 (4.5%) |
| Pneumonia | 141 (13.6%) | 23 (9.6%) | 22 (9.5%) | 29 (12.4%) | 67 (19.9%) |
| Acute respiratory symptoms | 275 (26.5%) | 54 (22.6%) | 62 (26.8%) | 61 (26.2%) | 98 (29.2%) |
| Fractures | 297 (28.6%) | 60 (25.1%) | 61 (26.4%) | 66 (28.3%) | 110 (32.7%) |
| Subdural hematoma | 13 (1.3%) | n<5 | n<5 | n<5 | 6 (1.8%) |
| Bleeding events | 232 (22.3%) | 46 (19.2%) | 42 (18.2%) | 49 (21.0%) | 95 (28.3%) |
| Thrombocytopenia | n<5 | n<5 | 0 (0%) | 0 (0%) | n<5 |
| Constipation | 502 (48.3%) | 84 (35.1%) | 98 (42.4%) | 118 (50.6%) | 202 (60.1%) |
| **Chronic events^a^** |  |  |  |  |  |
| Depression | 228 (21.9%) | 42 (17.6%) | 51 (22.1%) | 59 (25.3%) | 76 (22.6%) |
| Anxiety disorders | 165 (15.9%) | 33 (13.8%) | 43 (18.6%) | 46 (19.7%) | 43 (12.8%) |
| Obsessive-compulsive disorder | 16 (1.5%) | n<5 | 6 (2.6%) | 5 (2.1%) | n<5 |
| Dementia | 113 (10.9%) | 28 (11.7%) | 23 (10.0%) | 19 (8.2%) | 43 (12.8%) |
| Hydrocephalus | 19 (1.8%) | n<5 | n<5 | n<5 | 13 (3.9%) |
| Epilepsy | 78 (7.5%) | 9 (3.8%) | 7 (3.0%) | 17 (7.3%) | 45 (13.4%) |
| Communication and speech problems | 51 (4.9%) | 9 (3.8%) | 11 (4.8%) | 7 (3.0%) | 24 (7.1%) |
| Hypertension | 308 (29.6%) | 72 (30.1%) | 78 (33.8%) | 54 (23.2%) | 104 (31.0%) |
| Chronic renal impairment | 18 (1.7%) | n<5 | 6 (2.6%) | n<5 | 6 (1.8%) |
| Asthma | 60 (5.8%) | 11 (4.6%) | 12 (5.2%) | 12 (5.2%) | 25 (7.4%) |
| Liver failure/hepatic impairment/hepatitis | 8 (0.8%) | n<5 | n<5 | n<5 | n<5 |
| Gastrointestinal events | 379 (36.5%) | 71 (29.7%) | 81 (35.1%) | 84 (36.1%) | 143 (42.6%) |
| Thyroid disorders (hyper or hypothyroid) | 39 (3.8%) | 8 (3.3%) | 10 (4.3%) | 6 (2.6%) | 15 (4.5%) |
| Bone marrow disorders | 49 (4.7%) | 15 (6.3%) | 14 (6.1%) | 10 (4.3%) | 10 (3.0%) |
| Cancer | 90 (8.7%) | 18 (7.5%) | 18 (7.8%) | 17 (7.3%) | 37 (11.0%) |
| **Treatments** |  |  |  |  |  |
| Antipsychotic use | 497 (47.8%) | 70 (29.3%) | 108 (46.8%) | 132 (56.7%) | 187 (55.7%) |
| Tetrabenazine use | 209 (20.1%) | 43 (18.0%) | 42 (18.2%) | 58 (24.9%) | 66 (19.6%) |
| Antidepressants and mood stabilizers | 747 (71.9%) | 139 (58.2%) | 177 (76.6%) | 175 (75.1%) | 256 (76.2%) |
| Antiepileptic medications | 268 (25.8%) | 40 (16.7%) | 47 (20.3%) | 65 (27.9%) | 116 (34.5%) |
| Dopaminergic agents | 91 (8.8%) | 19 (7.9%) | 20 (8.7%) | 24 (10.3%) | 28 (8.3%) |
| PEG feeding | 111 (10.7%) | n<5 | 5 (2.2%) | 23 (9.9%) | 80 (23.8%) |

^a^Acute and chronic conditions were identified if individuals had at least one of the codes listed in the code categories in Appendix 1 using ICD-10 diagnosis codes in any position prior to index (1 January 2018), unless otherwise specified. Diagnosis and procedure codes were identified in the NPR and medication codes (ATC codes) were identified in the PDR. Cells with <5 cases were suppressed according to National Board of Health and Welfare advice.

^b^Secondary suppression due to low counts in other cells within that row.

Look-back duration: The time from the start of available data (immigration date or 1 January 2001, whichever later) until index date (1 January 2018).

Follow-up duration: The time from the index date to end of follow-up (date of death, emigration or 31 December 2018, whichever earliest), excluding the index date.

Due to Swedish confidentiality laws, categories observed in <5 people are marked as n<5.

ATC=Anatomical Therapeutic Chemical; HD=Huntington’s disease; ICD-10= International Classification of Diseases 10th Revision; NPR=Swedish National Patient Registry; PDR=Prescription Drug Registry; PEG=percutaneous endoscopic gastrostomy; SD=standard deviation.
