# Supplementary material for: Comorbidities and clinical outcomes in adult- and juvenile-onset Huntington’s disease: a study of linked Swedish National Registries (2002–2019)
Source: J Neurol. 2022 Oct 18;270(2):864–76. doi: 10.1007/s00415-022-11418-y (PMC9886595; doi:10.1007/s00415-022-11418-y)
Supplement: Supplementary file 2 — Supplementary file2 (DOCX 213 KB) [file 415_2022_11418_MOESM2_ESM.docx]

**Comorbidities and clinical outcomes in adult- and juvenile-onset Huntington’s disease: A study of linked Swedish National Registries (2002–2019)**

Hannah Furby,^1^ Suzanne Moore,^2^ Anna-Lena Nordstroem,^2^ Richard Houghton,^2^ Dimitra Lambrelli,^3^ Sophie Graham,^3^ Per Svenningsson,^4^ Åsa Petersén^5^

(1) Roche Products Ltd, Welwyn Garden City, UK

(2) F. Hoffmann-La Roche Ltd, Basel, Switzerland

(3) Evidera, London, UK

(4) Karolinska Institutet, Stockholm, Sweden

(5) Translational Neuroendocrine Research Unit, Department of Experimental Medical Science, Medical Faculty, Lund University, Lund, Sweden

**Online Resource 1**

**Supplementary File: Full medication and treatment code list**
